# Thyroid hypogenesis is associated with a novel *AKT3* germline variant that causes megalencephaly and cortical malformation

**DOI:** 10.1038/s41439-022-00197-7

**Published:** 2022-06-03

**Authors:** Jun Mori, Tatsuji Hasegawa, Yosuke Miyamoto, Kazumasa Kitamura, Hidechika Morimoto, Takenori Tozawa, Ritsuko Kimata Pooh, Tomohiro Chiyonobu

**Affiliations:** 1 Department of Pediatrics, Kyoto Prefectural University of Medicine, Kyoto, Japan; 2 Division of Pediatric Endocrinology and Metabolism, Children’s Medical Center, Osaka City General Hospital, Osaka, Japan; 3 Fetal Brain Center, Fetal Diagnostic Center, CRIFM Prenatal Medical Clinic, Osaka, Japan; 4 Clinical Laboratory, Ritz Medical Co., Ltd., Osaka, Japan; 5 Department of Molecular Diagnostics and Therapeutics, Graduate School of Medical Science, Kyoto Prefectural University of Medicine, Kyoto, Japan

**Keywords:** Central nervous system infections, Thyroid diseases

## Abstract

The molecular mechanisms involved in thyroid organogenesis have not been fully elucidated. We report a patient with a de novo germline *AKT3* variant, NM_005465.7:c.233A > G, p.(Gln78Arg), who presented with congenital hypothyroidism in addition to typical *AKT3*-related brain disorders. The report of this patient contributes to delineating the associated yet uncertain endocrine complications of this *AKT3* disease-causing variant.

Primary congenital hypothyroidism (CH) is the most common form of neonatal endocrine disease. Although recent advances in genetic technologies have identified CH-causing variants in several genes, the molecular mechanisms involved in thyroid organogenesis have not been fully elucidated^1^.

AKT proteins are a family of serine-threonine kinases that are central molecules of the phosphatidylinositol-3-kinase signaling pathway. They play critical roles in key cellular functions, such as cell cycle progression, proliferation, apoptosis, and metabolism^2^. AKT3 is expressed at high levels in the brain and endocrine tissues, especially in the thyroid. Recently, the clinical characteristics of *AKT3*-related brain disorders have been reported^3^. Somatic (mosaic) activating mutations in *AKT3* cause focal or segmental brain malformations, such as focal cortical dysplasia and hemimegalencephaly, whereas germline-activating mutations cause megalencephaly and diffuse bilateral cortical malformations^3^. Given the major role of AKT3 in cellular signal transduction, it is understandable that mutations in this gene may cause systemic disease. However, endocrine disorders have not been reported, except for hypoglycemia in a few patients^3,4^. Here, we report a case of a patient with a de novo germline *AKT3* variant, NM_005465.7:c.233A > G, p.(Gln78Arg), who presented with CH in addition to typical *AKT3*-related brain disorders.

A 39-year-old female was referred to our prenatal diagnosis center at 27 gestational weeks for fetal macrocephaly with cortical maldevelopment, as indicated by transvaginal 3D neurosonographic findings. Fetal magnetic resonance imaging (MRI) revealed that the fetus had asymmetrical megalencephaly with cortical dysplasia (Fig. 1A). Genomic DNA was extracted from amniotic fluid cells and parental lymphocytes using the DNeasy Blood & Tissue Kit (Qiagen, Hilden, Germany). Trio-based targeted exome sequencing was performed with the TruSight One Expanded Sequencing Panel (6704 genes) according to the manufacturer’s instructions (Illumina Inc., CA, USA). The prepared library was sequenced in 151 bp paired-end mode on an Illumina MiSeq (Illumina Inc., CA, USA). Sequencing reads were aligned and processed as previously reported^5^. Plausible variants were assessed according to the American College of Medical Genetics (ACMG) guidelines^6^. We identified a de novo heterozygous variant in *AKT3*, NM_005465.7:c.233A > G, p.(Gln78Arg), which was confirmed by Sanger sequencing (Fig. 1B). As this variant was not recorded in the public variant database, we concluded that this is a novel variant that is likely pathogenic (PS2, PM2, PP2, and PP3) based on ACMG guidelines. At 35 gestational weeks, 1500 mL amniotic fluid was drained because of polyhydramnios. The patient was delivered by cesarean section at 37 gestational weeks. His birth weight, height, and head circumference were 3529 g (+2.50 standard deviation (SD)), 49.0 cm (+1.24 SD), and 43.5 cm (+8.19 SD), respectively. He had a 1 min Apgar score of 2 and a 5 min Apgar score of 5. Soon after birth, he was intubated because of respiratory distress. The patient was treated with dopamine, dobutamine, and hydrocortisone to maintain blood pressure. His condition was stable, and he was extubated on the fifth day after birth. Sanger sequencing confirmed the presence of a heterozygous *AKT3* c.233A > G variant in peripheral blood lymphocytes. MRI of the brain showed asymmetrical megalencephaly with bilateral polymicrogyria (MEG-PMG) (Fig. 2A, B), which are typical presentations of germline-activating *AKT3* variants. The newborn screening revealed elevated levels of thyroid-stimulating hormone (TSH), which suggested hypothyroidism. On the 12th day after birth, a thyroid function test was performed. The results showed the elevation of TSH (1841 µIU/mL) and low levels of thyroid hormones (FT4 0.06 ng/dL, FT3 0.45 pg/mL), leading to the diagnosis of primary hypothyroidism. The breadth (cm), depth (cm), and length (cm) of the bilateral thyroid lobes were measured using thyroid ultrasound (right lobe: 0.57, 0.34, 0.92; left lobe: 0.85, 0.19, 1.22) (Fig. 2C). Thyroid volume was calculated using the following formula: thyroid volume (mL) = length (cm) × breadth (cm) × depth (cm) × π/6^7^. The volumes of the right thyroid and left thyroid were 0.093 mL (range (mL): 0.3–1.4) and 0.103 mL (range (mL): 0.4–1.3), respectively. Thyroid scintigraphy using ^99m^Tc pertechnetate did not reveal any functional thyroid tissue (Fig. 2D). Consequently, he was diagnosed with congenital hypothyroidism due to thyroid hypogenesis. L-T4 supplementation was initiated, and his blood levels of thyroid hormone normalized.

**Fig. 1:**
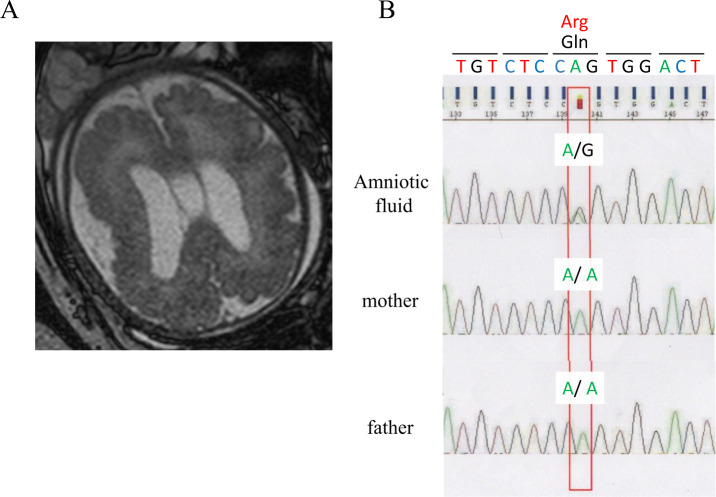
Fetal MRI findings in a patient with de novo heterozygous variant in the AKT3 gene. **A** Fetal magnetic resonance imaging (MRI) revealed that the fetus had asymmetrical megalencephaly with cortical dysplasia. **B** The chromatogram represents a *de novo* heterozygous c.233A > G (p.Gln78Arg) variant in the *AKT3* gene.

**Fig. 2:**
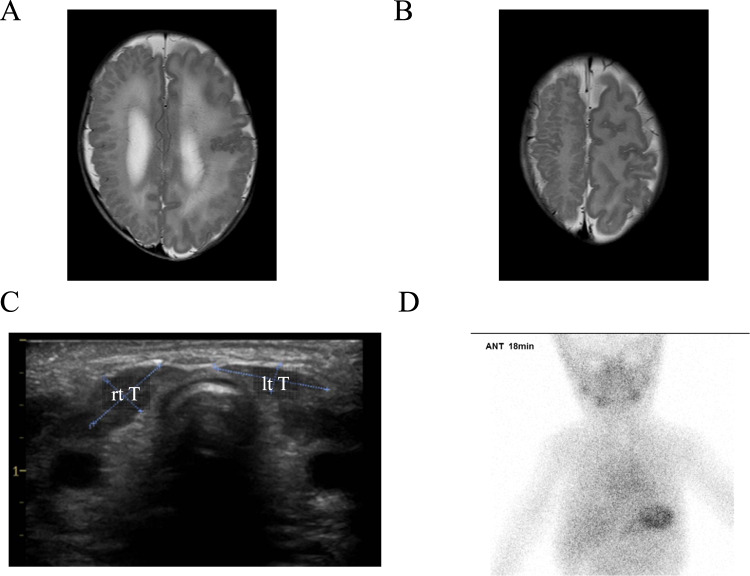
Images of the patient. **A, B** MRI of the brain showed asymmetrical megalencephaly with bilateral polymicrogyria. **C** Thyroid ultrasound examination revealed that both the right and left thyroid lobes were small. **D** Thyroid scintigraphy by ^99m^Tc pertechnetate showed no functional thyroid gland.

We report the case of a male patient with a novel *AKT3* variant, p.Gln78Arg, which localizes to the pleckstrin homology domain. Most previously reported germline *AKT3* missense variants causing MEG-PMG are within the pleckstrin homology domain or the catalytic kinase domain and result in elevated kinase activity^3^. Although functional studies to confirm the pathogenic effect of this variant has not been performed, the presence of typical brain malformations strongly indicates abnormal AKT3 function. He also presented with thyroid dysgenesis. His initial thyroid function test clearly showed primary hypothyroidism, and a thyroid ultrasound examination revealed that his thyroid was small. In addition, thyroid scintigraphy showed no functional thyroid glands. Thus, he was diagnosed with congenital hypothyroidism due to thyroid hypogenesis. To the best of our knowledge, this is the first report to describe a case of an *AKT3*-related brain disorder associated with thyroid hypogenesis.

The prevalence of thyroid dysgenesis is approximately one in 5000 live births. In Japan, most of these cases have ectopic thyroid (73%), while the rest have thyroid agenesis (12%) and thyroid dysgenesis (15%)^8^. The pathogenesis of thyroid dysgenesis remains unknown. Thyroid dysgenesis is generally considered sporadic. However, the frequency of familial cases is more than 15 times that of the general population, indicating that genetic factors could be associated with thyroid dysgenesis^9^. There are some transcription factors involved in thyroid genesis. Hhex is ubiquitously expressed in the foregut endoderm and drives the budding process. Fgf2 and Bmp4, which are derived from the cardiogenic mesoderm, induce thyroid progenitors from the foregut endoderm^10^. Thyroid progenitors express Nkx2-1 and Pax8 and regulate folliculogenesis. FOXE1 is expressed in thyroid follicular cells and plays an important role in thyroid development. Consequently, variants related to these transcription factors induce thyroid dysgenesis. No likely pathogenic variants of these genes related to thyroid dysgenesis were detected in this patient.

AKT is a serine/threonine-protein kinase that is involved in multiple cellular processes, such as glucose metabolism, apoptosis, cell proliferation, transcription, and cell migration. There are three isoforms of AKT: AKT1, AKT2, and AKT3. AKT3 is a candidate gene for thyroid dysgenesis and dysfunction, as AKT3 is highly expressed in the thyroid gland^11^. Iwadate et al. showed that NKX2-1 acts on thyroid development via AKT signaling in a mouse model, suggesting the possibility that the phosphorylation of AKT is associated with early stages of thyroid differentiation^12^. The detailed mechanism by which AKT3 is involved in the development of the thyroid gland is unknown. Reactive oxygen species (ROS) induce apoptosis in embryonic tissue, and a fine balance in ROS generation is required for embryonic and fetal development during the gestational period. In addition, ROS plays a pivotal role in the development and function of the thyroid^13^. The double knockout of *AKT1* and *AKT2* in mouse embryonic fibroblasts decreases ROS levels, suggesting that AKT increases ROS production^14^. Based on the evidence mentioned above, accelerated AKT activity due to *AKT3* mutations might increase the production of ROS, resulting in the inhibition of thyroid organogenesis.

In summary, the report of this patient contributes to delineating the associated yet uncertain endocrine complication of this *AKT3* disease-causing variant. However, there is the possibility of the incidental occurrence of hypothyroidism due to other causative genes for thyroid genesis that were not covered by the panel sequencing. To elucidate the relationship between *AKT3* variants and thyroid dysgenesis, further cases are needed.

## HGV database

The relevant data from this Data Report are hosted at the Human Genome Variation Database at 10.6084/m9.figshare.hgv.3195.

## References

[1] Targovnik HM, Scheps KG, Rivolta CM (2020). Defects in protein folding in congenital hypothyroidism. Mol. Cell Endocrinol..

[2] Toker A, Marmiroli S (2014). Signaling specificity in the Akt pathway in biology and disease. Adv. Biol. Regul..

[3] Alcantara D (2017). Mutations of AKT3 are associated with a wide spectrum of developmental disorders including extreme megalencephaly. Brain.

[4] Nellist M (2015). Germline activating AKT3 mutation associated with megalencephaly, polymicrogyria, epilepsy and hypoglycemia. Mol. Genet. Metab..

[5] Pooh RK (2021). Fetal megalencephaly with cortical dysplasia at 18 gestational weeks related to paternal UPD mosaicism with *PTEN* mutation. Genes (Basel)..

[6] Richards S (2015). ACMG laboratory quality assurance committee. Genet Med..

[7] Perry R, Hollman A, Wood A, Donaldson M (2002). Ultrasound of the thyroid gland in the newborn: normative data. Arch. Dis. Child Fetal Neonatal Ed..

[8] Narumi S, Muroya K, Asakura Y, Adachi M, Hasegawa T (2010). Transcription factor mutations and congenital hypothyroidism: systematic genetic screening of a population-based cohort of Japanese patients. J. Clin. Endocrinol. Metab..

[9] Castanet M (2001). Nineteen years of national screening for congenital hypothyroidism: familial cases with thyroid dysgenesis suggest the involvement of genetic factors. J. Clin. Endocrinol. Metab..

[10] Nilsson M, Fagman H (2017). Development of the thyroid gland. Development.

[11] Camargo RY (2018). Histopathological characterization and whole exome sequencing of ectopic thyroid: fetal architecture in a functional ectopic gland from adult patient. Int. J. Endocrinol..

[12] Iwadate M, Takizawa Y, Shirai YT, Kimura S (2018). An in vivo model for thyroid regeneration and folliculogenesis. Lab Invest..

[13] Li M (2022). Nicotinamide nucleotide transhydrogenase mutation analysis in Chinese patients with thyroid dysgenesis. Am. J. Med. Genet A..

[14] Nogueira V (2008). Akt determines replicative senescence and oxidative or oncogenic premature senescence and sensitizes cells to oxidative apoptosis. Cancer Cell..

